# Cessation of the Revista Medico Quirurjica Y Dentistica

**Published:** 1869-09

**Authors:** 


Cessation of the Revista Medico Quirurjica y Dentistica.—The proprietors of the *Revista Medico Quirurjica y Dentistica* announce the suspension of their journal. This is due solely to the political troubles now existing in the Spanish West Indies.

Publication will be resumed, it is hoped, in January, 1870.

Nearly every Medical Journal in the United States has exchanged with the *Revista;* and for this kindly recognition of their effort, the editors desire to express their thanks.

